# maxRatio improves the detection of samples with abnormal amplification profiles on QIAgen’s artus HIV-1 qPCR assay

**DOI:** 10.12688/f1000research.25738.1

**Published:** 2020-08-24

**Authors:** Luigi Marongiu, Eric B. Shain, Marianna Martinelli, Matteo Pagliari, Heike Allgayer

**Affiliations:** 1 Department of Experimental Surgery, University of Heidelberg, Medical Faculty in Mannheim, Mannheim, Baden-Wuttenberg, 68167, Germany; 2 Grove Street Technology LLC, Grove Street Technology LLC, Glencoe, Illinois, 60022, USA; 3 Department of Medicine and Surgery, University of Milano Bicocca, Monza, Italy, 20900, Italy; 4 Department of Diagnostics in Animal Health, Istituto Zooprofilattico Sperimentale delle Venezie, Legnaro, Veneto, 35020, Italy

**Keywords:** HIV, qPCR, quantification, maxRatio, viral load.

## Abstract

**Background:** Accurate viral load (VL) determination is paramount to determine the efficacy of anti-HIV-1 therapy. The conventional method used, fit-point (FP), assumes an equal efficiency in the polymerase chain reaction (PCR) among samples that might not hold for low-input templates. An alternative approach, maxRatio, was introduced to compensate for inhibition in PCR.

**Methods:** Herein, we assessed whether maxRatio could improve VL quantification using 2,544 QIAgen artus HI virus-1 RT-PCR reactions. The assay’s standard dilutions were used to build external standard curves with either FP or maxRatio that re-calculated the VLs.

**Results:** FP and maxRatio were highly comparable (Pearson’s ρ=0.994, Cohen’s  κ=0.885), and the combination of the two methods identified samples (n=41) with aberrant amplification profiles.

**Conclusions:** The combination of maxRatio and FP could improve the predictive value of the assay.

## Introduction

Infection with HIV-1 accounts for a global prevalence of 38 million cases and a one million deaths yearly ^1^. An accurate viral load (VL), typically carried out by quantitative polymerase chain reaction (qPCR), is pivotal for addressing the efficacy of antiviral therapies ^2^. The threshold level of detection for the HIV-1 (VL) has been reported in the range 20–44 viral genomic copies per milliliter (c/mL) ^3, 4^.

qPCR data are usually analyzed by the fit-point (FP) method, which assumes equal amplification efficiency between samples ^5^. However, anomalies in the background fluorescence at low template input, can affect the quantification ^6– 8^. An alternative method, maxRatio, was introduced to overcome these issues ^9^. It has been reported that maxRatio conferred a marginal increase in assay accuracy over FP ^10, 11^.

FP provides only a quantification cycle (Cq) value, which is then used to calculate VL. MaxRatio, instead, gives two parameters: one associated with the reaction’s efficiency (MR) and one equivalent to, albeit distinct from, Cq. These two parameters can be linked to bestow a quantitative cycle (FCNA) compensated for inhibition.

In the present work, we aimed to determine whether maxRatio could improve the determination of HIV-1 VL. We compared the quantification of HIV-1 VL computed by FP and maxRatio on a dataset generated with the QIAgen artus HIV assay, which has a reported limit of detection of 35.5 c/mL, and we showed that maxRatio could pinpoint samples with abnormal amplification profiles.

## Methods

### Dataset

The amplification data (see *Underlying data* ^12^) obtained with the QIAgen artus HI Virus-1 RT-PCR kit were collected by the Public Health England Clinical Microbiology and Public Health Laboratory, Addenbrooke’s Hospital, Hills Road, Cambridge CB2 0QW, UK, during the year 2016. All data were anonymized before use. The reactions were subdivided into clinical samples, control dilutions (CDs), and non-template controls (NTCs). The CDs were based on known dilutions of *in vitro* transcribed HIV-1 RNA provided by the artus kit, corresponding to 405, 4,050, 40,500, and 405,000 c/mL.

### Data analysis

The FP method generated the Cq by registering the fractional cycle where the fluorescence passed the threshold of 0.2 units. The maxRatio transform of the amplification data and determination of the cut-offs were computed as previously described ^9, 10^. Different operators visually inspected the reaction’s profiles and classified each reaction as either passed or failed. Using R v.3.6, linear models (standard curves, SC) were built on the CDs and applied to calculate the copy numbers according to the formula 10 ^( *x* – *b*)/ *m*^ where *x* is the quantitative cycle (either Cq or FCNA), *b* and *m* are the intercept and slope, respectively, of the linear models ^13^. VL correlation was obtained with the Pearson product-moment coefficient ρ ^14^ and agreement between methods was tested with the Cohen’s κ ^15^; both are reported with their 95% confidence interval (CI).

## Results

**Figure 1.**
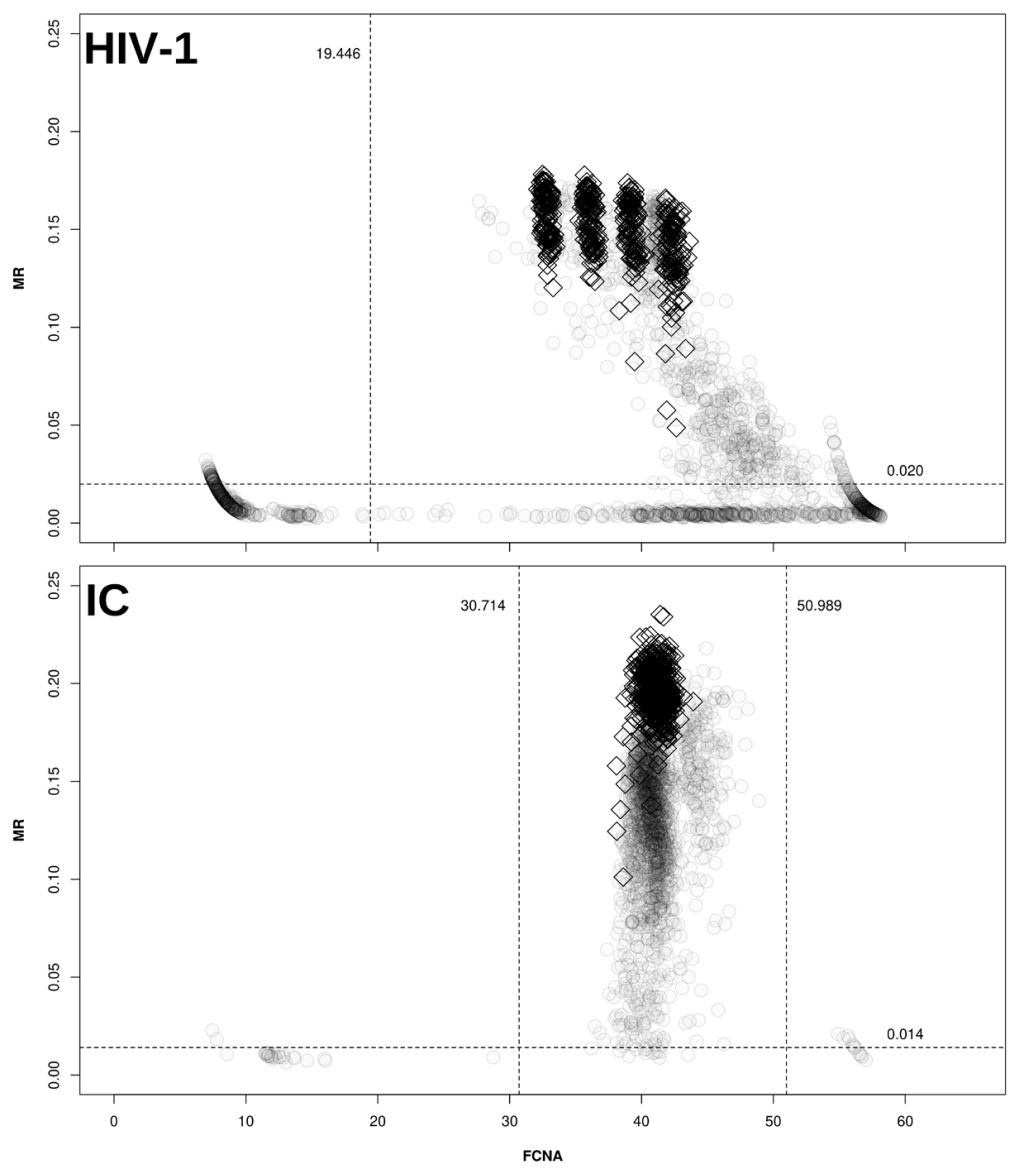
Cut-offs for maxRatio. Clinical samples (○) and CDs (◊) are plotted on the maxRatio plane; the numbers report the obtained cut-offs for MR (horizontal lines) and FCNA (vertical lines). A. MR/FCNA pairs for the HIV-1 target. To note how the CDs form four distinct clusters, corresponding to the different standard dilutions. B. MR/FCNA pairs for the IC target. To note that the data form a single cloud because the IC input was virtually the same for all reactions. The presence of two outlier groups at low and high FCNA values required to instantiate two cut-offs.

The present dataset was derived from 122 individual *artus* HIV-1 runs, corresponding to 2,544 reactions (480 CDs, 122 NTCs and 1,931 clinical samples). The cut-offs obtained by EM analysis were multiplied by 2.7 to generate the values used to filter the *maxRatio* data, as depicted in Figure 1.

**Figure 2.**
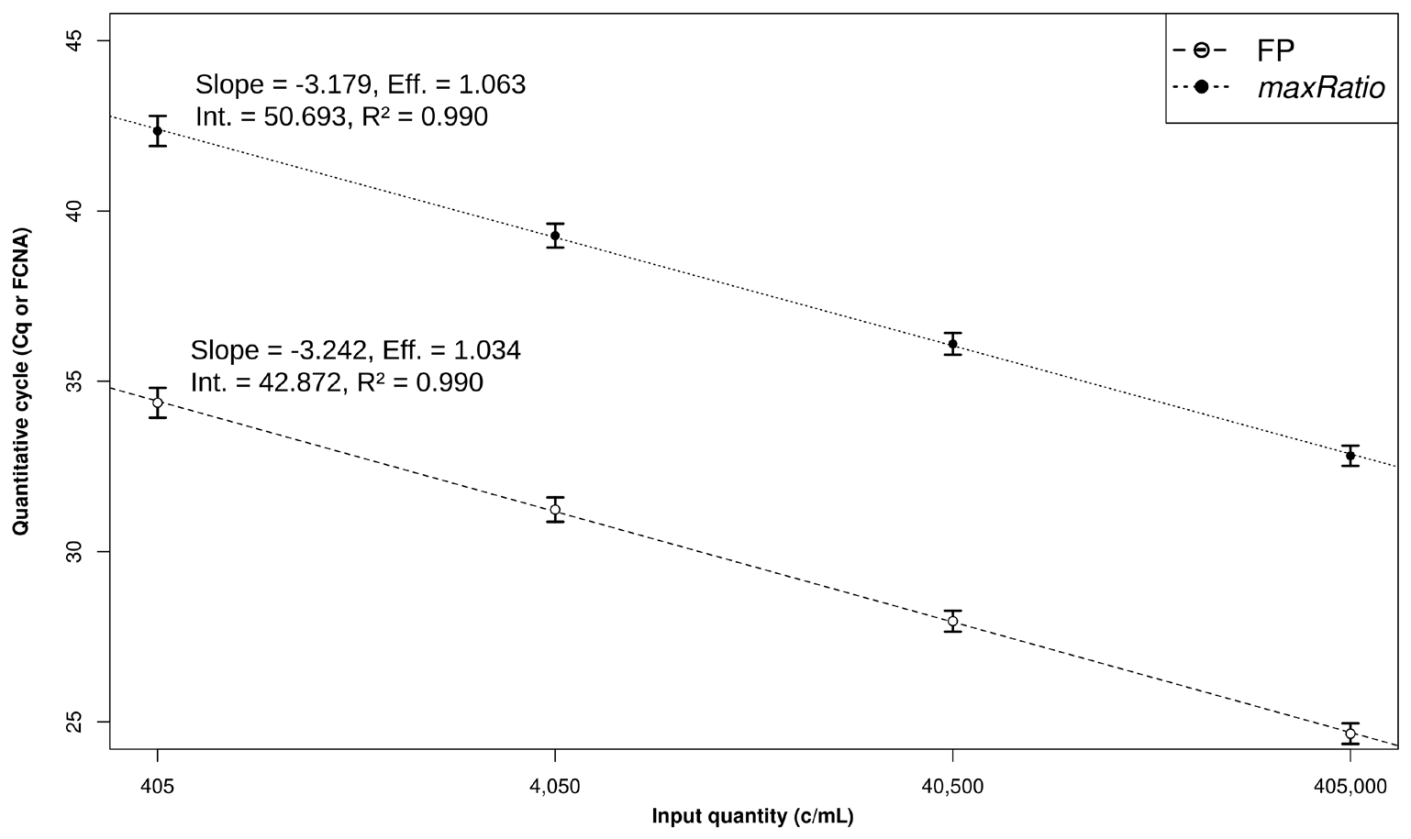
Linear models and CD quantification. Development of the linear models. The Cq (○) and FCNA (●) were used to build SCs for FP (dashed line) and maxRatio (dotted line). The dots and bars represent the mean and standard deviation of the data, respectively. The characteristics of the models are reported.

**Figure 3.**
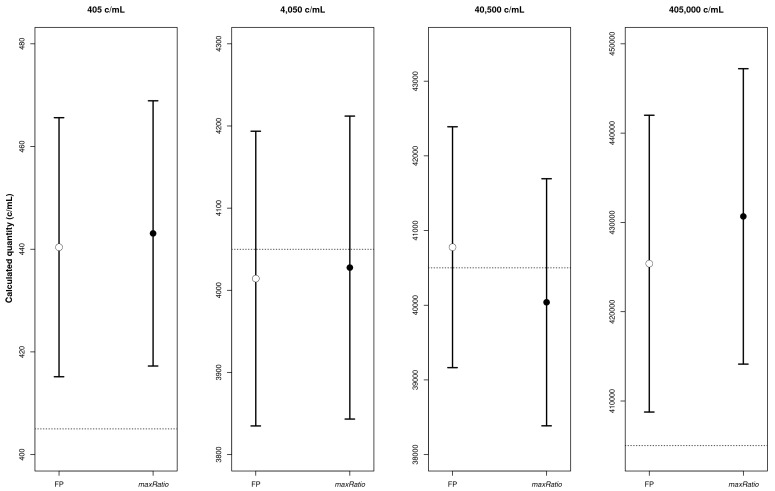
CD quantification. The four panels represent the calculated copy numbers obtained using FP (○) or maxRatio (●) for each dilution. The dots and bars represent the mean and the 95% CI of the data, respectively.

**Figure 4.**
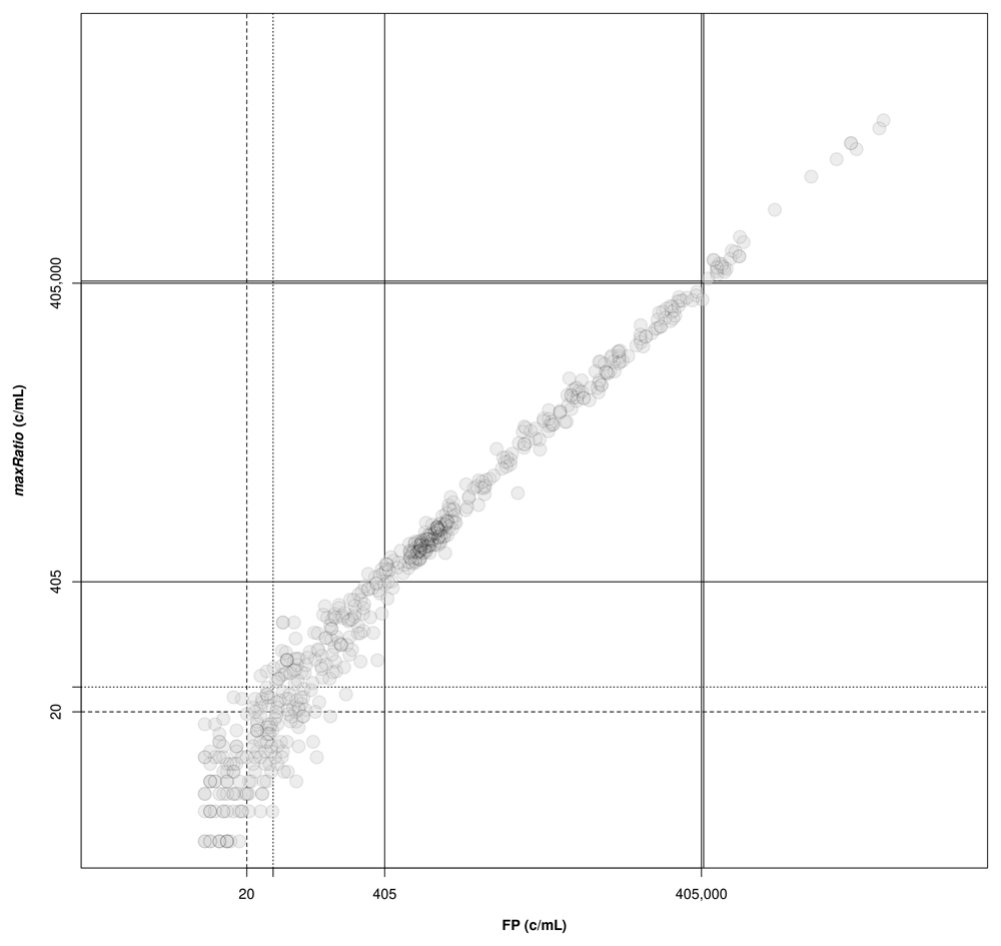
Correlation of VL obtained by FP and maxRatio. The external standard curves were used to calculate the VL for the clinical samples. The following thresholds are depicted: lower (solid lines) and upper (double solid lines) limits of the quantification range; limit of detection of HIV-1 diagnostic in general (dashed line) and artus HI Virus-1 assay in particular (dotted line).

The CDs were used to build SCs ( Figure 2) that quantified both the CDs ( Table 1 and Figure 3) and the clinical samples ( Figure 4). Overall, the VLs obtained with the two methods were very strongly correlated (ρ = 0.994, 95% CI: 0.993-0.994) and the agreement in the stratification of the reactions into reactive and non-reactive was almost perfect (κ = 0.885, 95% CI: 0.863-0.907). Both methods identified 307 (15.9%) and 28 (1.5%) samples within and above the quantification range 405–405,000 c/mL (ρ = 0.988, 95% CI: 0.985-0.991 and ρ = 0.992, 95% CI: 0.982-0.996, respectively), and 1,571 (81.3%) below this range (ρ = 0.844, 95% CI: 0.829-0.858).

**Table 1.** Comparison of copy numbers for the control dilutions. The mean VL is reported together with the 95% CI (calculated), the difference between the calculated and the expected concentration (difference), and the result of the t-test (p-value). Statistical significance is represented by * and ** for values below 0.05 and 0.01, respectively. The copy numbers are given in c/mL.

Dilution	FP			*maxRatio*		
Expected	Calculated	Difference	p-value	Calculated	Difference	p-value
405	440 (415-466)	35	0.006**	443 (417-469)	38	0.004**
4,050	4,014 (3,835-4,194)	36	0.694	4,028 (3,843-4,212)	22	0.811
40,500	40,777 (39,164-42,389)	277	0.735	40,039 (38,385-41,693)	–461	0.582
405,000	425,384 (408,765-442,002)	20,383	0.017*	430,673 (414,132-447,215)	25,673	0.003**

**Figure 5.**
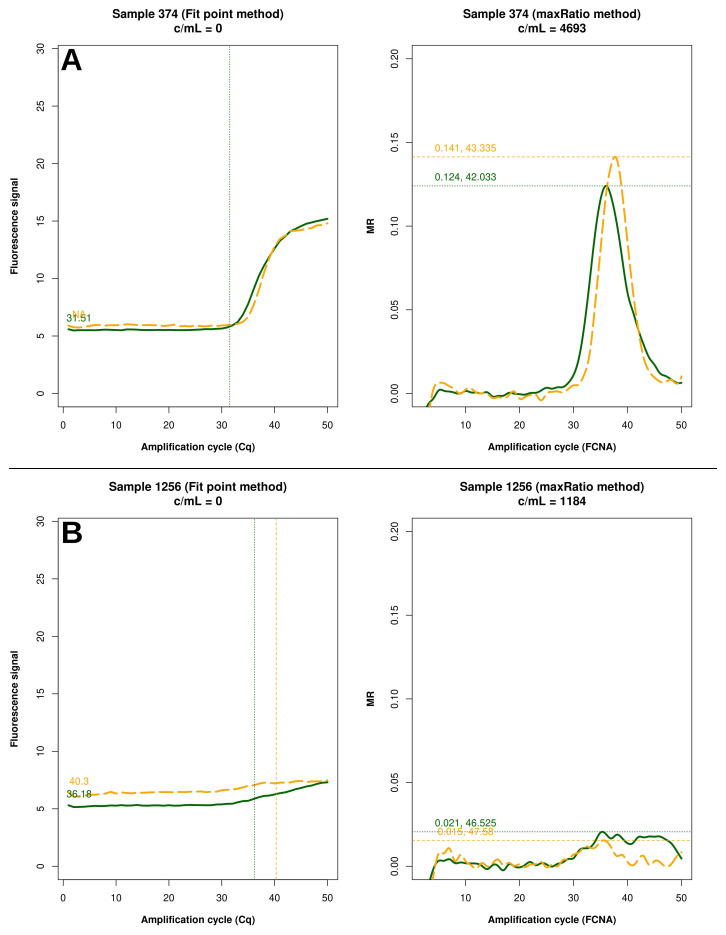
Samples not quantified only by FP. Example of raw amplification profiles for the samples whose FP did not provide a VL. The panels displays the FP (left) the maxRatio (right) transforms of the amplification data. The solid line represents the HIV-1 template and the dashed line the IC template. ( **A**) The majority of the samples had a proper IC profile but the output was undetermined. ( **B**) The minority of the samples showed low target inputs.

**Figure 6.**
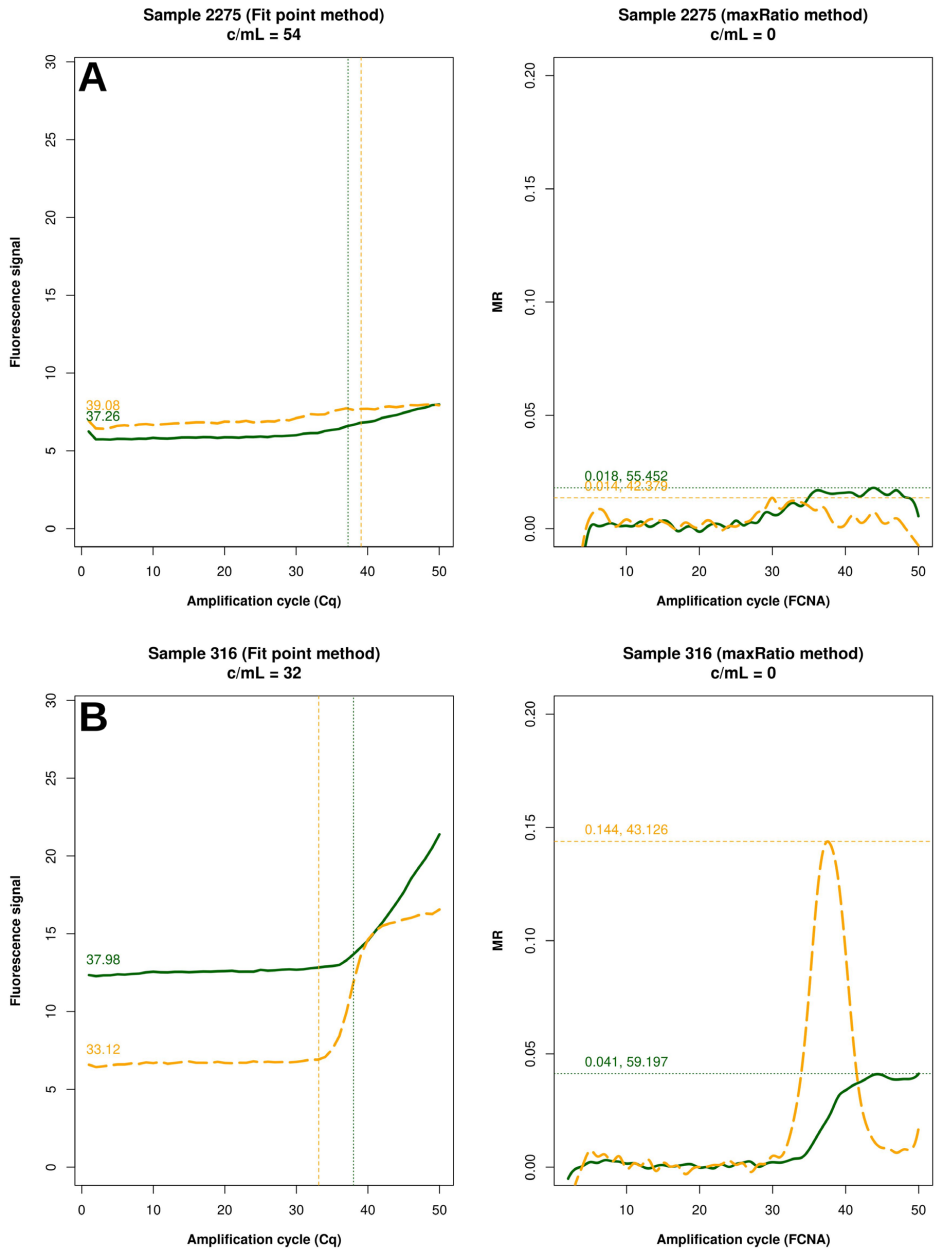
Samples not quantified only by maxRatio. Example of raw amplification profiles for the samples whose maxRatio did not provide a VL. The panels displays the FP (left) the maxRatio (right) transforms of the amplification data. The solid line represents the HIV-1 template and the dashed line the IC template. ( **A**) The majority of the samples had abnormal IC profiles that were overlooked by FP. ( **B**) The minority of the samples showed proper amplification profiles, but the calculation provided fractional VLs that were rounded to zero.

FP identified 22 reactions above the limit of detection of 20 c/mL, while maxRatio failed the quantification; conversely, maxRatio identified 18 reactions below 20 c/mL while FP quantified them above this level. Visual inspection of the amplification profiles of the reactions failed by FP showed that 10 of them (43.4%) had a proper sigmoid outlook for the internal control (IC) signal that, however, was discarded by the FP (as exemplified in Figure. 5A). In contrast, the others had a low signal for either HIV-1 or IC recovered by maxRatio ( Figure 5B). Conversely, 15 (83.3%) of the reactions failed by maxRatio showed either a low IC or HIV-1 input ( Figure 6A), whereas the FCNA of the remainder of the reactions produced fractional VL that were rounded to 0 c/mL ( Figure 6B).

## Discussion

The purpose of the present work was to assess the potential benefits of maxRatio in determining HIV-1 VL given its inherent compensation of PCR inhibition. Contrary to our expectation, the SCs we built with either FP or maxRatio were virtually the same. Both methods gave SD VLs significantly divergent from the expected copy numbers at the lower and upper CDs, and maxRatio was, in general, more discrepant from the expected copy numbers than FP. Even concerning the samples’ quantification, the two methods produced essentially the same VLs.

The main difference between the two methods was in terms of sample’s reactivity. By accepting the reactions identified as non-reactive by FP, but reactive by maxRatio, there would have been 18 false-negative results. Conversely, 15 samples identified as non-reactive by maxRatio showed aberrant IC that raised quality control, rather than false-positive, issues overlooked by FP.

The current use of maxRatio is to confirm the reactivity determined by FP on the Abbott m2000rt platform. Our data supported this combination as the most effective approach for screening purposes. Samples in disagreement between FP and maxRatio would require further assessment that could reduce the workload involved in issuing the results and minimize the risk of providing false results.

The present work had some limitations. Firstly, the sample size was small. Secondly, the CDs were prepared by diluting the control samples provided in the kit, but the actual concentration was not measured. Finally, we did not have access to the actual issued results; thus, we could not confirm the official VL values.

In conclusion, we compared FP and maxRatio in providing HIV-1 VL. Contrary to our expectations, maxRatio did not give a better quantification than FP, but combining the two methods could minimize issuing false results.

## Data availability

### Underlying data

Harvard Dataverse: artusHIV_amplificationData. https://doi.org/10.7910/DVN/0QQNPF

This project contains the following underlying data:

- amplificationDataRaw.tab. (Raw amplification data.)
- viralLoads.tab. (Raw viral loads data.)

Data are available under the terms of the Creative Commons Zero "No rights reserved" data waiver (CC0 1.0 Public domain dedication).
