# Supplementary material for: maxRatio improves the detection of samples with abnormal amplification profiles on QIAgen’s artus HIV-1 qPCR assay
Source: F1000Res. 2021 Aug 31;9:1030. Originally published 2020 Aug 24. [Version 3] doi: 10.12688/f1000research.25738.3 (PMC8442116; doi:10.12688/f1000research.25738.3)
Supplement: Peer review history

# Peer review: Reviewer response for version 3

**DOI:** 10.5256/f1000research.58856.r93097

**Published:** 2021-09-14

**Authors:** Joel Tellinghuisen

**Affiliations:** 1 Department of Chemistry, Vanderbilt University, Nashville, TN, USA

With minor exceptions, I accept the author’s explanations and clarifications. Regarding the data, I wish I had saved what I downloaded initially but I did not. I recall clicking somewhere on the left side of the download page, and in two attempts, I got nothing sensible. I *was* able to get the data now, though they seem to be space-separated rather than csv; and the file would be much smaller if the fluorescence data didn’t have 13 figures after the decimal point!

 My suggestion of just specifying a minimal *plateau* level was based on the observation that most of the questionable rxs never actually grow, and those that grow only a little for late cycles ultimately give 0 copies anyway. I did not intend this to mean anything about threshold levels.

 I agree that my thinking was flawed when I suggested that the MR cycle would roughly equal the FDM: Maximum first difference indeed does not equal maximum ratio. My comment about instrument dependence was also wrong, as indeed the ratios will not change with scaling. On the other hand, two identical growth profiles superimposed on different baselines will give different MRs, and that would seem to make their FCNAs different.  In this connection, is the conversion from FCN to FCNA used here as given in eq 2 in ref 9? That eqn is FCNA = FCN – log2(MR). When I apply that, for example, to the green curve in Fig. 5B (MR), I get FCNA ~ 36 – log2(0.021) = 41.6, which is much smaller than the given 46.525. Should that eqn perhaps have a factor of 2 with the log2 term?

Is the work clearly and accurately presented and does it cite the current literature?

Partly

If applicable, is the statistical analysis and its interpretation appropriate?

Partly

Are all the source data underlying the results available to ensure full reproducibility?

No

Is the study design appropriate and is the work technically sound?

Partly

Are the conclusions drawn adequately supported by the results?

Partly

Are sufficient details of methods and analysis provided to allow replication by others?

No

Reviewer Expertise:

NA

I confirm that I have read this submission and believe that I have an appropriate level of expertise to confirm that it is of an acceptable scientific standard.

# Peer review: Reviewer response for version 3

**DOI:** 10.5256/f1000research.58856.r93096

**Published:** 2021-09-01

**Authors:** Emiliano Panieri

**Affiliations:** 1 Department of Physiology and Pharmacology, Faculty of Pharmacy and Medicine, Sapienza University of Rome, Rome, Italy

I have no further comments

Is the work clearly and accurately presented and does it cite the current literature?

Yes

If applicable, is the statistical analysis and its interpretation appropriate?

Yes

Are all the source data underlying the results available to ensure full reproducibility?

Yes

Is the study design appropriate and is the work technically sound?

Yes

Are the conclusions drawn adequately supported by the results?

Yes

Are sufficient details of methods and analysis provided to allow replication by others?

Yes

Reviewer Expertise:

Oncology, cellular and molecular biology

I confirm that I have read this submission and believe that I have an appropriate level of expertise to confirm that it is of an acceptable scientific standard.

# Peer review: Reviewer response for version 2

**DOI:** 10.5256/f1000research.57778.r89311

**Published:** 2021-07-13

**Authors:** Joel Tellinghuisen

**Affiliations:** 1 Department of Chemistry, Vanderbilt University, Nashville, TN, USA

    The authors use a method apparently first described in their ref 9 (2008), which was coauthored by the 2 ^nd^ author of this work. This maxRatio method seems to be a useful tool for automated screening of very large numbers of qPCR reaction profiles for non-detects, though it is not clear to me that it is much better than, for example, just setting a low limit on acceptable plateau levels, perhaps combined with an upper limit on acceptable *C _q_* values, beyond which the template number would round to zero. I did not see any comparisons of this sort either here or in ref 9. And I am not well enough acquainted with the various instruments to know what other possible tools there are for this kind of discrimination. However, it does appear to me that the title is not quite a truthful description of the work, since the authors note in Results that the MR method and the “fixed point” (FP) method with which they compare it gave nearly identical “stratification of the reactions into reactive and non-reactive.” And then early in the Discussion (and again in the final paragraph of the Conclusion), they acknowledge that the two methods gave virtually identical standard curves (SC). On the other hand, it is not clear to me how the discrimination was done in the FP method. This may have been by manual inspection (bottom of 1 ^st^ column, p 3), in which case the MR method would still be advantageous. (But, as noted, the “inspection” could also be automated.)

             The near-agreement in the 2 standard curves is not surprising, because the FCNA is essentially a first-derivative maximum (FDM), shifted slightly from the use of finite differences and even more by the correction –log2(MR) (which is not mentioned here but is given in eq 2 in ref 9). Incidentally, this correction must only apply for a particular instrument and its fluorescence axis scale, because it clearly changes as the data are scaled. In any event, both *C _t_* (used here for *C _q_* in the FP method) and the FDM are legitimate *C _q_* markers, so they should indeed give similar SCs. In fact, *C _t_* is arguably the poorest *C _q_* marker, for reasons Spiess and I have explained, most recently in Biomol. Detect. Quantif. 17 (2019) 100084. ^1^ Thus, the use of, for example, a *relative* threshold obtained from a fit model that includes the baseline function, should give even better FP results.

             I wanted to check some of the authors’ results but unfortunately could not easily get the data from their referenced source (p 9 and ref 12). The Excel files seem not to be .csv, as labeled; the .tab files are text, but the application for opening them is not given (GitHub perhaps?). Nor is their content explained. I did attempt to reproduce their SCs in Figure 2, by digitizing the plotted data (WebPlotDigitizer, https://apps.automeris.io/wpd/). My results more or less confirmed theirs with one exception: My uncertainties for the back-calculated *N*_0_ values for FP in Table 1 were ~10% smaller than theirs for the first 3 concentrations but ~40% larger for the highest. The relative errors here depend on only the structure of the data, so the last should also have been small by ~10% (this discrepancy from digitization limitations). The larger error makes the discrepancy < 2 *s* for this case, removing one of the two values judged to be discordant.

 Some specific problems:

1. Some labels are not defined: EM (1 ^st^ para of Results), SD (5 ^th^ line of Discussion). And IC appears in Fig. 1 but isn’t defined until several paragraphs after Fig. 1 is mentioned.
2. I don’t understand the introductory sentence in the last para of Results. And if the 10 which had sigmoidal appearance were of 22, that would be 45.4%, not 43.4%.
3. While it is clear that the reaction profiles in Figs 5B and 6A should not give results, I don’t understand why those in 5A do not.

Is the work clearly and accurately presented and does it cite the current literature?

Partly

If applicable, is the statistical analysis and its interpretation appropriate?

Partly

Are all the source data underlying the results available to ensure full reproducibility?

No

Is the study design appropriate and is the work technically sound?

Partly

Are the conclusions drawn adequately supported by the results?

Partly

Are sufficient details of methods and analysis provided to allow replication by others?

No

Reviewer Expertise:

statistical data analysis

I confirm that I have read this submission and believe that I have an appropriate level of expertise to state that I do not consider it to be of an acceptable scientific standard, for reasons outlined above.

## References

[1] : qPCR data analysis: Better results through iconoclasm.*Biomol Detect Quantif*.2019;17: 10.1016/j.bdq.2019.10008410008410.1016/j.bdq.2019.10008431194178PMC6554483

# Response

**Published:** 2021-07-19

**Authors:** Luigi Marongiu

**Affiliations:** University Heidelberg (Medizinische Fakultat MA), Germany

Eric B. Shain is indeed the developer of maxRatio, as reported in Ref. 9. Shain and Marongiu collaborated to apply maxRatio in qPCR classification as either reactive or non-reactive in other works. The purpose of the present study was to assess whether maxRatio could provide more accurate copy numbers than the classical FP due to maxRatio's inherent capability to compensate for qPCR inhibition. Contrary to our expectations, maxRatio did not provide a significant improvement over FP. Hence we considered our results as "grey," and we embraced the F1000Research policy of welcoming "confirmatory and negative results, as well as null studies... irrespective of the perceived level of interest or novelty".

 Our title reflects the fact that maxRatio helped identify reactions that were classified as non-reactive by FP. Unsurprisingly, the classification of the majority of the reactions was similar between the two methods. The focus of the analysis was instead on the few disagreed reactions, which are also those where an operator might have more difficulties inspect. About half of the reactions classified as non-reactive by FP had a proper amplification profile, suggesting false-negative results. Conversely, this ratio was only about a fifth using maxRatio. Despite the small sample set, we believe that when applied to the vast volumes characteristic of diagnostic laboratories, such a minor improvement could still be beneficial.

 As reported in the Method section, the amplification profile of the individual reactions was visually inspected by a trained operator, and the operator classified each reaction as either reactive or non-reactive.  Conversely, classification by maxRatio was done directly by mathematical methods. The evaluation of the amplification profiles generated by maxRatio was done post hoc purposedly to compare the two methods.

 For the sake of brevity, we did not report the details of the FCNA's calculation. However, we referred to the original paper cited on maxRatio, as correctly noted by the reviewer, including the details of the expectation-maximization (EM) step reported in the first paragraph of the results section. We agree with the reviewer that the slope of the standard curves built with either FP or maxRatio should be similar. The focus of the work was not on the subtle differences between the standard curves based on the two methods but their applications.

 The driving force of this and the previous works on the application of maxRatio done by Shain and Marongiu was to avoid the definition of a baseline threshold level. Firstly, the guidelines for the threshold value are too generic to give universal outcomes. Secondly, the threshold value might not be the same for each PCR assay. Thirdly, in our experience, the threshold level is set without much mathematical consideration. Conversely, maxRatio provides an objective way to quantify the reactions that dispense the threshold level altogether. As the reviewer remarked in his cited paper (Biomol Detect Quantif 2019;17:100084) the threshold level does not hold throughout the PCR while "most workers have taken [it] as a level near baseline where it is hoped that eq. 2 [associating the copy number to the efficiency of the reaction] remains valid". We agree with the conclusion of the author in his paper -- that is, that the absolute threshold is a poor choice -- because we believe that maxRatio allows overcoming its use. We disagree with the reviewer when he stated, "it is not clear to me that [maxRatio] is much better than, for example, just setting a low limit on acceptable plateau levels". If the reviewer meant to lower the threshold, there would be firstly the problem mentioned above of where to set the cut-off level, and secondly, there would be a trade-off between picking up more true positives at the expense of more false positives. This trade-off would require further determination of the impact of the analytical method on the sensitivity and specificity of the assay that (a) was beyond the scope of our work and (b) is skipped altogether by using maxRatio.

 Furthermore, we disagree with the reviewer when he remarked, "FCNA is essentially a first-derivative maximum (FDM), shifted slightly from the use of finite differences and even more by the correction –log2(MR)". In fact, maxRatio is not the same as FDM. maxRatio does not use differences. maxRatio consistently identifies a cycle number earlier and closer to the exponential portion of the amplification curve. In addition, maxRatio provides a second measure (MR) which is highly useful in evaluating reactive-nonreactive status. FCNA helps correct the quantitation of PCR results where the response is partially inhibited. FDM cannot do this. We also disagree with his statement, "This correction must only apply for a particular instrument and its fluorescence axis scale because it clearly changes as the data are scaled." maxRatio works by calculating ratios. It is entirely unaffected by multiplicative scaling effects.

 We loaded the datasets following the Harvard Dataverse's guidelines, essentially uploading a comma-delimited table. We quickly downloaded the original data simply by clicking on the "download" icon and selecting the download option. On downloading the data saved on Harvard Dataverse, the comma-separated values original file format)" can be opened directly with a spreadsheet application (in our case: LibreOffice Calc).

 On downloading the alternative version "tab-delimited", Harvard Dataverse automatically adds the extension .tab, but the file's layout remains comma-delimited, implying that the spreadsheet applications do not appropriately recognize such file format. We recommend downloading the dataset only in its original format.  Nevertheless, the procedure of data downloading is under the domain of Harvard Dataverse.

 We believe that the names of the fields in the datasets were self-explicatory, but we have now included a dictionary to supply more information.

 We assume that the discrepant back-calculated copy numbers might be due to the different approaches taken (actual calculation based on the quantification thresholds in our paper, digitalization applied by the reviewer).

# Peer review: Reviewer response for version 2

**DOI:** 10.5256/f1000research.57778.r88542

**Published:** 2021-06-29

**Authors:** Emiliano Panieri

**Affiliations:** 1 Department of Physiology and Pharmacology, Faculty of Pharmacy and Medicine, Sapienza University of Rome, Rome, Italy

The authors properly addressed all my previous requests. I am satisfied with their reply and I have no additional issues.

Is the work clearly and accurately presented and does it cite the current literature?

Yes

If applicable, is the statistical analysis and its interpretation appropriate?

Yes

Are all the source data underlying the results available to ensure full reproducibility?

Yes

Is the study design appropriate and is the work technically sound?

Yes

Are the conclusions drawn adequately supported by the results?

Yes

Are sufficient details of methods and analysis provided to allow replication by others?

Yes

Reviewer Expertise:

Oncology, cellular and molecular biology

I confirm that I have read this submission and believe that I have an appropriate level of expertise to confirm that it is of an acceptable scientific standard.

# Peer review: Reviewer response for version 1

**DOI:** 10.5256/f1000research.28406.r83772

**Published:** 2021-06-10

**Authors:** Emiliano Panieri

**Affiliations:** 1 Department of Physiology and Pharmacology, Faculty of Pharmacy and Medicine, Sapienza University of Rome, Rome, Italy

**General comment:**

 The paper from Marongiu *et al.* presents an alternative approach to the conventional use of fit-point (FP) or MaxRatio methods, which can be used to accurately determine the viral load of HIV-1 patients under experimental conditions wherein the amplification efficiency might vary across the samples, as in the case of low-copy number of viral RNA or in presence of PCR inhibitors. This is a clinically relevant issue since the VLs widely differ among HIV-1 infected individuals, potentially leading to issues of false-positive/negative results when the viremia is very low or the efficiency of PCR reactions is not uniform. It is therefore of utmost importance to improve not only the analytical sensitivity of qPCR methods but also their predictive value to avoid that a substantial fraction of HIV-1 patients will be incorrectly diagnosed, causing a negative impact on their prognosis and treatment monitoring. In this regard, the approach proposed by the authors, based on both FP and MaxRatio is straightforward and when correctly applied can represent a cost-effective alternative to the time-consuming and labor-intensive work required to validate false positive and false negative results. In general, the manuscript is well written, concise and presented in a clear, scientifically-sound way. The data are quite exhaustive, corroborated by appropriate statistical analysis and firmly support the authors’ conclusions.

 However, some minor changes will be required to improve the MS quality, which are listed below:

- The authors correctly indicate that the small-size of the tested samples represents a limitation to their study. Can also the authors discuss whether increasing the sample size is expected to proportionally increase the accuracy of the proposed method? In other words, in the authors’ opinion, is the number of tested samples expected to influence the predictive value of the FP + MaxRatio use? It might be useful to further elaborate this aspect in the discussion to help the readers to gain insights on this limitation.
- The authors should indicate which kind of t-test was used to assess the statistical significance of the data contained in Table 1 (i.e paired or unpaired, with or without other corrections such as Welch’s or Bonferroni’s) and which software was used to perform the statistical analysis.
- In the Results paragraph it is suggested to mitigate the following statement: ”…the agreement in the stratification of the reactions into reactive and non-reactive was almost perfect (κ = 0.885, 95% CI:0.863-0.907). ”. Instead, “noticeably robust” can be a good alternative.
- Few lines below it is suggested to slightly rearrange the sentence “…the FP identified 22 reactions above the limit of detection of 20 c/mL, while maxRatio failed the quantification” (i.e. while Max Ratio was unsuccessful).
- Few lines below it is suggested to substitute “sigmoid outlook” with “sigmoidal shape”.
- Few lines below it is suggested to change “…of the remainder of the reactions” into “…of the remaining reactions”.
- In the Figure 1 legend, the authors describe a panel A (MR/FCNA pairs for the HIV-1 target) and a panel B (MR/FCNA pairs for the IC) which are however absent in the Figure 1. Please add the correct indication or either refer to the panels as upper and lower.
- In the Figure 5-6 legend, it should be corrected “the panels displays” with “the panels display”.
- In the last three lines of the Discussion section please rephrase the sentence: “…maxRatio did not give a better quantification than FP, but combining the two methods could minimize issuing false results” into “maxRatio did not result in a better quantification than FP but when combined together these two methods could…”

Is the work clearly and accurately presented and does it cite the current literature?

Yes

If applicable, is the statistical analysis and its interpretation appropriate?

Yes

Are all the source data underlying the results available to ensure full reproducibility?

Yes

Is the study design appropriate and is the work technically sound?

Yes

Are the conclusions drawn adequately supported by the results?

Yes

Are sufficient details of methods and analysis provided to allow replication by others?

Yes

Reviewer Expertise:

Oncology, cellular and molecular biology

I confirm that I have read this submission and believe that I have an appropriate level of expertise to confirm that it is of an acceptable scientific standard.
